# How do differences in native language affect out-of-body experiences?

**DOI:** 10.3389/fpsyg.2024.1350980

**Published:** 2024-06-06

**Authors:** Hirotaka Uchitomi, Yuma Yamamoto, Kishiko Ueno, Yuko Nomura, Yoshihiro Miyake

**Affiliations:** ^1^Department of Computer Science, Tokyo Institute of Technology, Yokohama, Japan; ^2^Faculty of Liberal Arts and Sciences, Tokyo City University, Setagaya, Japan; ^3^Faculty of International Liberal Arts, Juntendo University, Bunkyo, Japan

**Keywords:** out-of-body experiences, OBE, native language, cognitive phenomena, Japanese, English, sense of embodiment

## Abstract

Out-of-body experiences are scientifically inducible cognitive phenomena attracting global attention due to their application in the Metaverse and medical care. Despite previous studies suggesting that one’s native language influences one’s cognition, the out-of-body experiences of humans with different native languages have not been investigated separately. This study replicated an experiment from a 2007 study to investigate whether differences in native language affect the ability to have scientifically induced out-of-body experiences. A total of 19 age-matched native English and Japanese speakers completed the experiment in two blocks. Thereafter, their experiences were evaluated using questionnaires, and their responses were compared. Importantly, no significant differences between the English and Japanese native-speaker conditions were found. The results showed that out-of-body experiences were induced similarly in both groups, suggesting that people can have out-of-body experiences as a response to similar stimuli, regardless of their native language. However, differences in participants’ introspective reports suggested that their experiences may differ qualitatively, possibly, due to the different linguistic backgrounds. The elucidation of the mechanisms of science-assisted out-of-body experiences that consider different cultural and cognitive characteristics, such as native language, could lead to the investigation of their applications in the borderless Metaverse and medicine.

## 1 Introduction

Out-of-body experiences (OBEs) are scientifically inducible cognitive phenomena that have recently attracted global attention due to their application in the Metaverse (Moon and Han, 2022) and medical care (Szczotka and Wierzchoń, 2023). In the Metaverse, individuals can embody avatars, computer-generated representations of themselves, and interact with others using avatars that resemble real individuals (Petrigna and Musumeci, 2022). The Metaverse is often connected with immersive experiences in virtual worlds, resulting in a growing interest in virtual reality (VR) and augmented reality systems and applications that provide lifelike multimodal sensory experiences. Regardless of native language or national borders, plans to utilize these technologies for various social implementations and applications is becoming widespread.

An OBE is defined as a cognitive experience in which the awake person views their physical body from outside (Brugger et al., 1997; Blanke et al., 2004). OBEs were originally reported in clinical conditions that interfere with normal brain functioning, such as strokes, partial-onset seizures (epilepsy), and drug abuse (Grüsser and Landis, 1991; Brugger et al., 1997; Brugger, 2002; Blanke et al., 2004). However, this psychological phenomenon can occur just as well in the normal population (Blackmore, 1982, 1984), suggesting the possibility that our self-body perceptions are not always with our actual bodies themselves. Moreover, OBEs can occur during wakefulness and sleep, including in persons initiated from sleep paralysis (Cheyne and Girard, 2009). Surprisingly, it has become possible to induce OBEs scientifically in recent years. Ehrsson (2007) reported that this illusory experience can also be induced in healthy participants. In such instances, individuals experience the perceptual illusion of their center of consciousness, or “self,” being outside their body and see it from another person’s perspective. This illusion is an effect of having the sensation of localization in the body realized through a perceptual process, that is, by combining a visual perspective with multisensory stimulation. Moreover, this OBE has been reported to be a highly reproducible cognitive phenomenon in follow-up studies (Guterstam and Ehrsson, 2012), and brain functions related to OBE have been elucidated using fMRI (Guterstam et al., 2015). However, the OBEs of persons with different native languages have not been investigated sufficiently.

Previous studies have reported on the relationship between native language and human cognitive phenomena. One previous study (Flecken et al., 2014) used eye-tracking to investigate how language influences attention to motion event recognitions. It compared the speakers of two different native languages in non-verbal tasks. Their results contributed to the language-and-thought debate by examining grammatical concepts. Another previous study (Athanasopoulos et al., 2015) focused on the extent to which language affects the process that people use to make sense of objects and events around them by classifying them into identifiable categories. Their findings suggested that different languages caused their speakers to behave differently. However, the relationship between OBEs as cognitive phenomena and the native language of the persons experiencing these phenomena has not been clarified.

Therefore, this study aimed to examine and report whether differences in native language affect OBEs. From the above background, our working hypothesis is that OBEs are perceptual illusions based on a combination of multisensory stimuli; consequently, regardless of differences in people’s native languages, OBEs are elicited in the same way based on human being cognitive functions related to illusions. However, we also hypothesize that people with different native languages report different verbal content when interpreting OBEs verbally. To test this, we followed the methodology reported by Ehrsson (2007) for his Experiments #1 and #2, with age-matched native English speakers and native Japanese speakers as the participants. Self-evaluations of OBEs were collected using the same questionnaire methods as those used by Ehrsson (2007); thereafter, we compared the results.

## 2 Materials and methods

To investigate the effect of different native languages on OBEs, age-matched native English speakers (number: 13, age: 27.4 ± 3.2, height: 169.1 cm ± 10.1, weight: 67.2 kg ± 10.3) and native Japanese speakers (number: 9, age: 21.2 ± 1.1, height: 168.0 cm ± 7.2, height: 168.0 cm ± 7.2, weight: 57.0 kg ± 9.4) were recruited to participate in this study (see Table 1 for the sample’s demographic information). There were no differences in height, weight, or sitting height between the two populations.

**Table 1:** Participants’ demographic characteristics.

Item	Native English speakers	Native Japanese speakers
Number of participants (male, female)	13 [M:7, F:6]	9 [M:7, F:2]
Age (years)	27.4 (±3.2)	21.2 (±1.1)
Height (m)	169.1 (±10.1)	168.0 (±7.2)
Sitting height (m)	118.5 (±3.2)	118.3 (±2.7)
Body weight (kg)	67.2 (±10.3)	57.0 (±9.4)
History of residence in Japan (foreigners only)	2.6 (±1.3)	–
Common European Framework of Reference for Languages (CEFR) [Level: A1-C2]	(Native language)	A2 level: 2 people B1 level: 5 people B2 level: 2 people
Japanese Language Proficiency Test (JLPT) [Level: N1-N5]	N1 level: 2 people N3 level: 4 people N4 level: 1 people N5 level: 6 people	(Native language)

The study was conducted in conformity with the Declaration of Helsinki and was approved by the Ethics Committee of the Tokyo Institute of Technology (Permit No.: 2021217). Written informed consent was obtained from all participants. Three native English speakers were excluded from the analyses: one who experienced a system error during the experiment and two who had had extensive experience with VR games, which could have led to decreased relevance of the illusion and thus less responsiveness (Fribourg et al., 2021).

**Figure 1:**
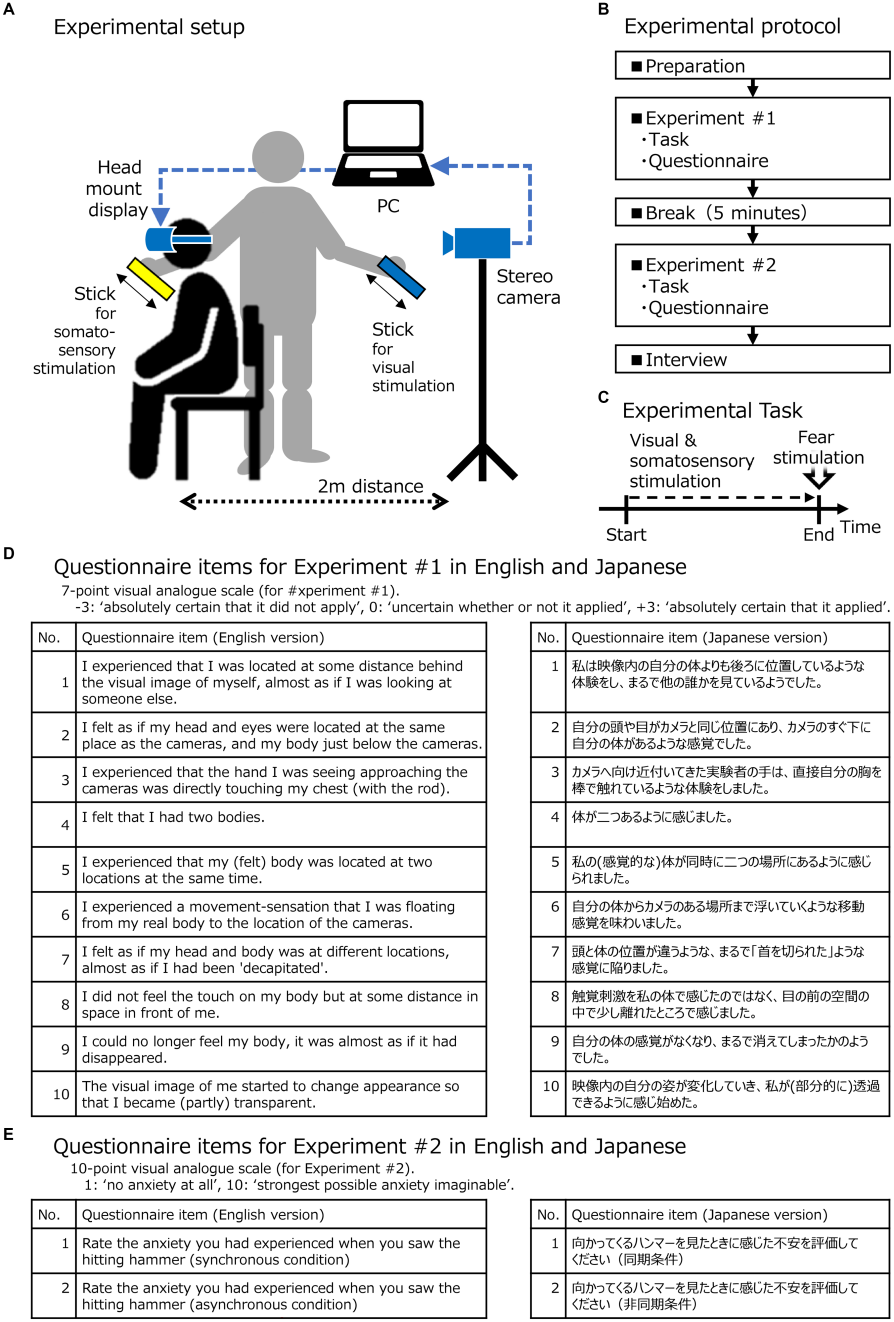
Organization of Blocks #1 and #2. **(A)** The experimental system, setup, and task used to induce the out-of-body experience illusion. **(B)** The experimental protocol of Block #1 and Block #2, including preparation, breaks, and an interview. **(C)** The experimental task with timeline. **(D)** Questionnaire items for Block #1 in English and Japanese scored on a 7-point visual analog scale (−3: “absolutely certain that it did not apply”; 0: “uncertain whether or not it applied”; +3: “absolutely certain that it applied”); **(E)** Questionnaire items for Block #2 in English and Japanese scored on a 10-point visual analog scale (1: ‘no anxiety at all;’ 10: ‘strongest possible anxiety imaginable’).

The experimental setup was arranged as shown in Figure 1. Each subject sat on a chair and wore a head-mounted display (HMD) connected to a stereo camera and positioned 2 m behind their back at the same height as their eyes. The stereo camera consisted of two monocular red-green-blue cameras (left and right) and was wired to a PC, which transmitted the images to the HMD using the Unity programming language (Unity Software Inc.) and an experimental software developed using C#; the image from the left video camera was displayed on the left-eye display, and the image from the right camera was displayed on the right-eye display. Thus, the person saw their own back from the perspective of the person sitting behind them in the stereoscopic view. The experimental software had two modes: the synchronous mode, in which the stereo camera images were displayed on the HMD in real-time, and the asynchronous mode, in which the stereo camera images were displayed on the HMD with a 0.5-s delay. Experimental Block #1 used only the synchronous mode, whereas experimental Block #2 used both the synchronous and asynchronous modes.

The stimulation method of the experiment was applied according to the hypothesis of Ehrsson’s (2007) study, in which illusions were induced from a first-person perspective, combining visual and tactile information. Sticks for visual and somatosensory stimulation were used in both blocks. Additionally, to examine the participants’ emotional responses to their illusory body being “injured,” a visual fear stimulus was provided by having the experimenter swing a hammer at the stereo camera as if he would hit it; the participants saw the hammer swinging down toward their faces via the HMD.

In this study, both Blocks #1 and #2 consisted of a task and a structured questionnaire. There was a 5-min break between the two blocks, after the completion of which, unstructured interviews were conducted with participants. The experimental protocol, setup, and questionnaire items are illustrated in Figure 1.

### 2.1 Experimental block #1

**Figure 2:**
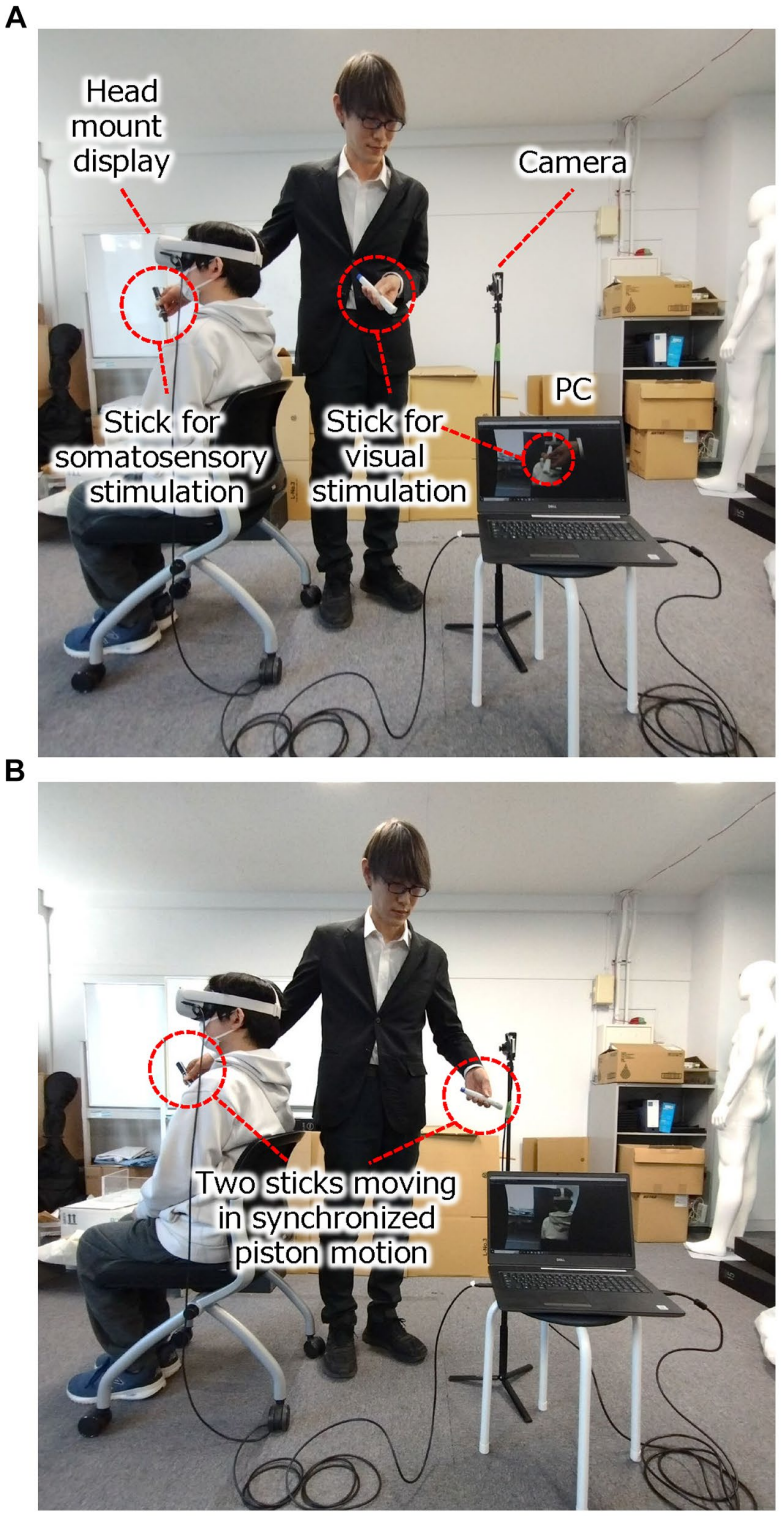
Experiment scenery of the illusion of out-of-body experience and the experimental result of Block #2. **(A)**. The experimental setup used to induce the illusion of an out-of-body experience. **(B)** The synchronized visual and somatosensory stimuli are given to the experimental participants. (Written informed consent was obtained from the participants in the figures for the publication of identifying information/images in an online open-access publication).

Block #1 consisted of simultaneous stimuli of a visual and somatosensory kind, as shown in Figure 1. The experimental task started with the participant sitting on the chair and observing the stereo camera images using the HMD. The experimental software was set to synchronous mode, and stereo camera images were presented to the participants in real-time. The experimenter stood right next to the participants (in their view) and used two plastic sticks to simultaneously touch the chest of the invisible person and the chest of the “illusory body” as shown in Figure 2 (written informed consent was obtained from the participants in the figures for the publication of identifying information/images in an online open-access publication), moving the visual-stimulation stick downward from the center of the stereo camera in front of it. To the participants wearing the HMD, it appeared as if the stick was tapping their chest. Thus, the participants synchronously felt the visual and somatosensory stimuli, and this simultaneous stimulation was administered continuously for 2 min. It should be noted that Block #1 included only this experimental condition of applying synchronized simultaneous stimuli of visual and somatosensory kind to participants.

Afterward, a visual fear stimulus was applied. The experimenter swung a hammer at the stereo camera as if he would hit it. The participants saw the hammer swinging down toward their faces via the HMD. This stimulus was only applied once to each participant.

After completing the experimental task, participants were asked to answer a structured questionnaire, presented in Figure 1, in which they either affirmed or denied cognitive effects. This questionnaire consisted of 10 items (Q1-Q10), each scored on a visual analog scale from −3 to +3, where −3 meant “absolutely certain that it did not apply,” 0 meant “uncertain whether or not it applied,” and + 3 meant “absolutely certain that it applied.” Items Q1-Q3 were designed to capture the experience of the illusion, while Q4-Q10 were unrelated to OBEs and served as controls. The native English speakers answered the English questionnaire and the native Japanese speakers answered the Japanese questionnaire.

The questionnaire results for the first block were analyzed in two ways: first, the average scores of Q1-Q3 and Q4-Q10 were calculated for each participant. These were then compared between native English and Japanese speakers using a significance test for linear regression with a generalized linear model (GLM). This GLM consisted of the gamma distribution and the invers link function. The objective variable was the average score, and the explanatory variables were NATIVE-LANGUAGE Factor (NL-Factor) and QUESTIONNAIRE-SCORE Factor (QS-Factor). NL-Factor was related to the native language {“native English speaker,” “native Japanese speaker”}, and QS-Factor was related to the questionnaire items about cognitive effects {“Q1, 2, 3,” “Q4, 5, 6, 7, 8, 9, 10”}. This significance test was conducted by converting the scores from −3 to +3 into scores from 1 to 7 because of the GLM with the gamma distribution. In this way, we analyzed whether OBEs were induced among both linguistic groups. If an OBE was induced, the scores for “Q1, 2, 3” would have been significantly higher than those for “Q4, 5, 6, 7, 8, 9, 10.” If there was a difference in the induction of OBEs between native English and Japanese speakers, there would have been a significant difference between the two groups in the ratings of all the questionnaire items. Second, we compared the mean scores of the questionnaire results for each item from Q1 to Q10 between the two groups. Mann–Whitney’s U test was used for comparison.

### 2.2 Experimental block #2

Block # 2, just like Block # 1, included simultaneous visual and somatosensory stimuli and the same visual fear stimulus. However, the difference was the implementation of the asynchronous condition as a control condition. In this condition, the somatosensory and visual stimuli were no longer synchronized.

The duration of the visual and somatosensory stimuli in the experimental task was set randomly between 40 and 80 s. The task was performed six times for each participant: three times in the synchronous condition and three times in the asynchronous one. The order of the experimental tasks in both conditions was pseudo-randomized, and either (1,2,2,1,1,1,2) or (2,1,1,1,2,2,2,1) was used, where 1 indicates the synchronous condition, and 2 indicates the asynchronous one.

After completing all six experimental tasks, we administered a structured questionnaire (see Figure 1), which asked the participants to rate the anxiety they experienced when they saw the hammer swinging down toward their faces via the HMD, on a visual analog of a 10-point Likert scale. The items were numbered Q11 and Q12 for the synchronous and asynchronous conditions, respectively. On this scale, “1” meant “no anxiety at all,” and “10” meant “the worst possible anxiety imaginable.” Native English speakers rated the English questionnaire items, and native Japanese speakers rated the Japanese questionnaire items.

The questionnaire results for the second block were analyzed as follows: averages were calculated per participant for Q11 and Q12, and comparisons were made between the synchronous and asynchronous conditions in STIMULI-PATTERN Factor (SP-Factor). In addition, comparisons were made between synchronous and asynchronous conditions for native English and Japanese speakers in NL-Factor. A significance test for GLM was used for the comparisons. This GLM consisted of the poison distribution and the log link function. The objective variable was the score, and the explanatory variables were NL-Factor and SP-Factor, as defined above. Thus, we analyzed whether OBEs were induced among both groups. If an OBE was induced, the Q11 evaluation value for the synchronous condition would be significantly higher than the Q12 evaluation value for the asynchronous condition. If the groups differed in their ability to have OBEs, a significant difference was expected to be observed between Q11 and Q12 for the respective conditions.

### 2.3 Unstructured interviews

Unstructured interviews were conducted after the completion of all the blocks. The participants were asked, “Please describe, in as much detail as possible, what you experienced and felt during the experiment.” They were also given the following main points to discuss: “visual body versus actual body,” “how you felt when being tapped,” and “how you felt when you saw the hammer.” Instructions were given in English to native English speakers and in Japanese to native Japanese speakers. All participants responded freely in their native language.

Two video cameras and two integrated-chip recorders were used to record the interviews. All utterances in the interviews were transcribed and used for analysis.

## 3 Results

### 3.1 Result in block #1

**Figure 3:**
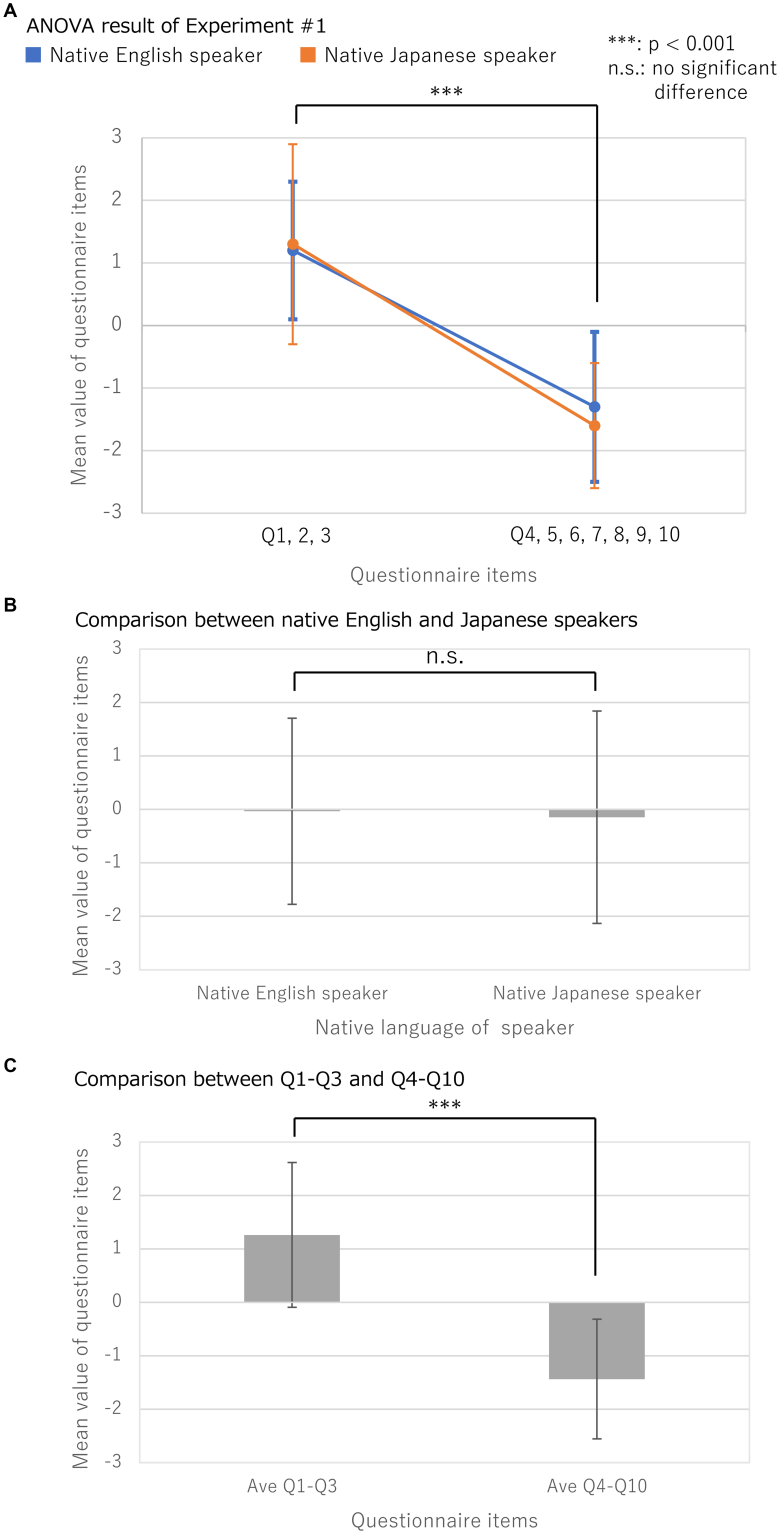
Experimental result of Block #1. **(A)** Mean values and standard deviations for questionnaire items after Block #1 a two-factor Mixed-Design Analysis of Variance (ANOVA) with and without correspondence, was used to analyze NL-Factor for native language {“native English speaker,” “native Japanese speaker”} and QS-Factor for the cognitive-effects questionnaire items {“Q1, 2, 3,” “Q4, 5, 6, 7, 8, 9, 10”} were compared; **(B)** Results of the comparison between native English and Japanese speakers; **(C)** Results of the comparison between Q1-Q3 and Q4-Q10.

The experimental results in Block #1, presented in Figure 3 and Tables 2, 3, showed a significant main effect for the QS-Factor, related to the cognitive-effects questionnaire items (*p* < 0.0001). Concurrently, the main effect of the NL-Factor was not significant (*p* = 0.9), neither was there an interaction between the QS-Factor and NL-Factor (*p* = 0.5). These results indicate that OBEs were induced similarly in both groups. Importantly, no significant differences were found between English and Japanese native speakers in the NL-Factor.

**Table 2:** Mean values and standard deviations for the questionnaire items after Block #1.

NL-Factor	QS-Factor	Mean (±SD)
Native English speakers	Q1, 2, 3	1.23 (±1.13)
	Q4, 5, 6, 7, 8, 9, 10	−1.30 (±1.25)
Native Japanese speakers	Q1, 2, 3	1.30 (±1.64)
	Q4, 5, 6, 7, 8, 9, 10	−1.59 (±1.01)
Native English speakers	–	−0.03 (±1.74)
Native Japanese speakers	–	−0.16 (±1.99)
–	Q1,2,3	1.26 (±1.35)
–	Q4,5,6,7,8,9,10	−1.44 (±1.12)

**Table 3:** Result of the significance test for linear regression with a generalized linear model (GLM) in Experimental Block #1.

	Dependent variable			
Predictors	Estimates	Confidence interval (CI)	*p*	
(Intercept)	1.21	1.16–1.27	<0.001	***
NL-Factor	1	0.94–1.06	0.943	n.s.
QS-Factor	1.2	1.09–1.32	<0.001	***
NL-Factor × QS-Factor	1.05	0.91–1.21	0.526	n.s.

NATIVE-LANGUAGE Factor (NL-Factor): {“native English speaker,” “native Japanese speaker”}. QUETIONNAIRE SCORE Factor (QS-Factor): {“Q1,2,3,” “Q4,5,6,7,8,9,10”}. This GLM consisted of the gamma distribution and the inverse link function. The objective variable was the average score, and the explanatory variables were NATIVE-LANGUAGE Factor (NL-Factor) and QUESTIONNAIRE-SCORE Factor (QS-Factor). NL-Factor was related to the native language {“native English speaker,” “native Japanese speaker”}, and SP-Factor was related to the questionnaire items about cognitive effects {“Q1, 2, 3,” “Q4, 5, 6, 7, 8, 9, 10”}. “***” means significant difference with *p* < 0.001; “n.s.” means non-significant difference.

**Figure 4:**
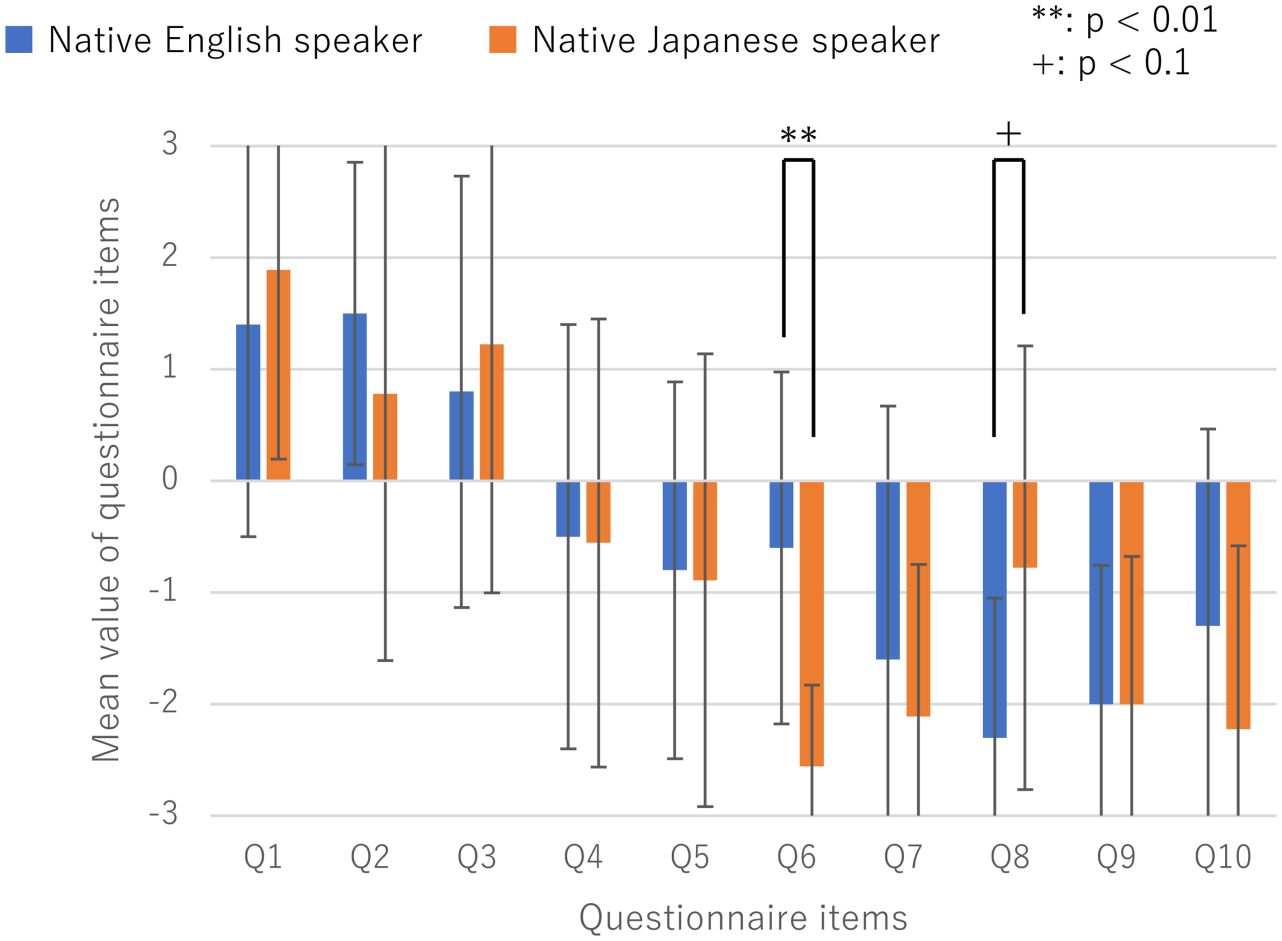
Mean values and standard deviations for each of the 10 questionnaire items regarding participants’ introspection of their OBEs to determine if there were differences between native English and Japanese speakers.

As an additional detailed examination of the experimental results, we analyzed whether there were differences between the native English and Japanese speakers for each of the 10 questionnaire items used in Block #1. The results are presented in Figure 4 and Table 4. Interestingly, among the questionnaire items, Q4–10 were unrelated to OBEs, and native English speakers scored significantly higher in Q6 than native Japanese speakers (*p* < 0.01). Q6 read: “I experienced a movement-sensation that I was floating from my real body to the location of the cameras.” Furthermore, native Japanese speakers had significantly higher scores in Q8 than native English speakers (*p* < 0.05). Q8 read: “I did not feel the touch on my body but at some distance in space in front of me.” These differences in introspective reports might be related to qualitative differences in OBEs between native English and Japanese speakers.

**Table 4:** The results of the statistical analyses on the differences between native English and native Japanese speakers for each question item after Block #1.

Questionnaire item no.	Native English speakers	Native Japanese speakers	*p*-value	
Q1	1.4 (1.9)	1.9 (1.7)	0.563	n.s.
Q2	1.5 (1.4)	0.8 (2.4)	0.422	n.s.
Q3	0.8 (1.9)	1.2 (2.2)	0.663	n.s.
Q4	−0.5 (1.9)	−0.6 (2.0)	0.951	n.s.
Q5	−0.8 (1.7)	−0.9 (2.0)	0.918	n.s.
Q6	−0.6 (1.6)	−2.6 (0.7)	0.003	^**^
Q7	−1.6 (2.3)	−2.1 (1.4)	0.566	n.s.
Q8	−2.3 (1.3)	−0.8 (2.0)	0.059	+
Q9	−2.0 (1.2)	−2.0 (1.3)	1.000	n.s.
Q10	−1.3 (1.8)	−2.2 (1.6)	0.256	n.s.

“^**^” means significant difference with *p* < 0.01; “^+^” means marginally significant difference with *p* < 0.1; “n.s.” means non-significant difference.

There were no significant differences in the responses given by the two groups to any of the other questionnaire items (*p* > 0.1).

### 3.2 Result in block #2

**Figure 5:**
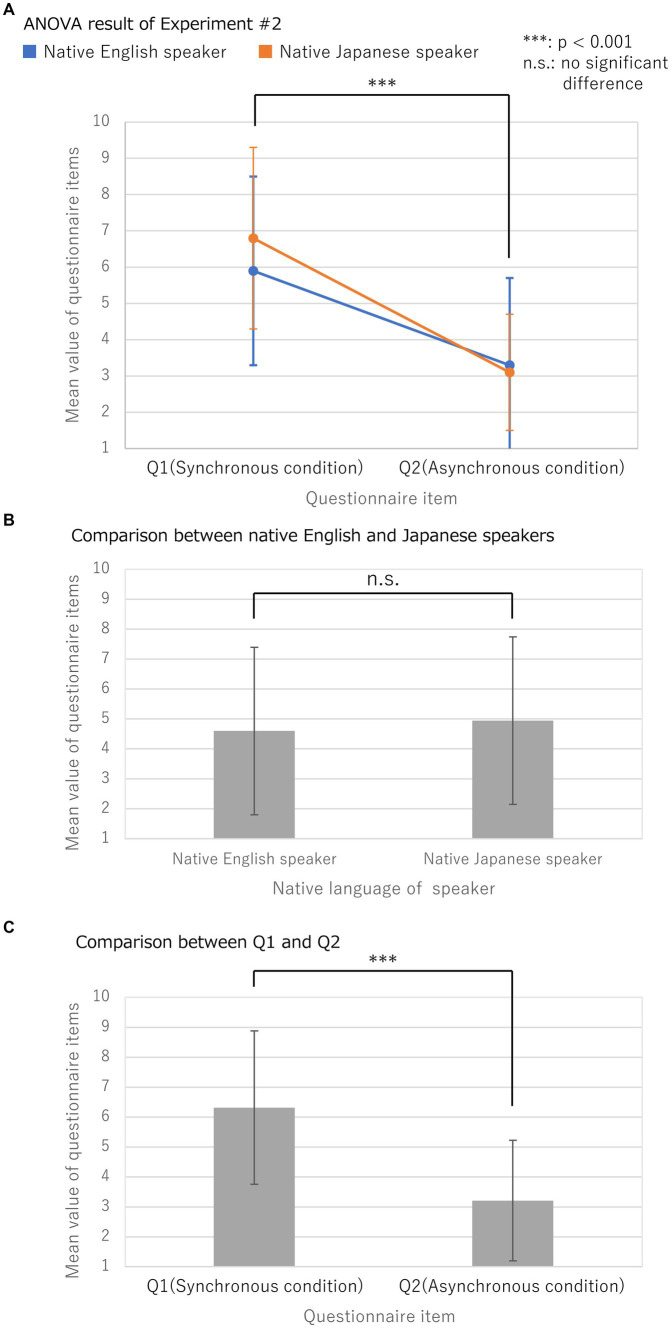
Experimental result of Block #2. **(A)** Mean values and standard deviations for questionnaire items after Block #2, a two-factor Mixed-Design Analysis of Variance (ANOVA) with and without correspondence, was used to analyze NL-Factor for native language {“native English speakers,” “native Japanese speakers”} and SP-Factor for the cognitive-effects questionnaire items {“Q1(Synchronous condition),” “Q2(Asynchronous condition)”} were compared; **(B)** Results of the comparison between native English and native Japanese speakers; **(C)** Results of the comparison between questionnaire items Q1 (synchronous condition) and Q2 (asynchronous condition) in Block #2.

The results in Block #2 are presented in Figure 5 and Tables 5, 6. The experimental results displayed in Figure 5 showed a significant main effect for the SP-Factor (*p* < 0.01). Subsequently, the NL-Factor’s main effect was found to be insignificant (*p* = 0.4). There was also no interaction between the SP-Factor and NL-Factor (*p* = 0.5). These results indicated that the OBEs were induced similarly in both groups. Moreover, no significant differences were found between English and Japanese native speakers in the NL-Factor.

**Table 5:** Mean values and standard deviations for questionnaire items after Block #2.

NL-Factor	SP-Factor	Mean (±SD)
Native English speakers	Q1 (Synchronous condition)	5.90 (±2.64)
	Q2 (Asynchronous condition)	3.30 (±2.41)
Native Japanese speakers	Q1 (Synchronous condition)	6.78 (±2.54)
	Q2 (Asynchronous condition)	3.11 (±1.62)
Native English speakers	–	4.60 (±2.80)
Native Japanese speakers	–	4.94 (±2.80)
–	Q1 (Synchronous condition)	6.32 (±2.56)
–	Q2 (Asynchronous condition)	3.21 (±2.02)

**Table 6:** Result of the significance test for linear regression with a generalized linear model (GLM) in Experimental Block #2.

	Dependent variable			
Predictors	Incidence rate ratios	Confidence interval (CI)	*p*	
(Intercept)	5.9	4.52–7.54	<0.001	***
NL-Factor	1.15	0.80–1.65	0.448	n.s.
SP-Factor	0.56	0.36–0.85	0.008	***
NL-Factor × SP-Factor	0.82	0.44–1.52	0.531	n.s.

NATIVE-LANGUAGE Factor (NL-Factor): {“native English speaker,” “native Japanese speaker”}. STIMULI-PATTERN Factor (SP-Factor): {“Q11” “Q12”}. This GLM consisted of the poison distribution and the log link function. The objective variable was the score, and the explanatory variables were NATIVE-LANGUAGE Factor (NL-Factor) and STIMULI-PATTERN Factor (SP-Factor). NL-Factor was related to the native language {“native English speaker,” “native Japanese speaker”}, and SP- Factor was related to the questionnaire items about cognitive effects {“Q11” “Q12”}. “^***^” means significant difference with *p* < 0.001; “n.s.” means non-significant difference.

### 3.3 Result in unstructured interviews

English native speakers were characterized by talking in detail about (1) the sensation of perspective and the body shifting toward the camera and (2) the sensation of the body floating or receding. The detailed results of these unstructured interviews are presented in the Supplementary data S1 and Supplementary Figure S2. Six of the 10 native English speakers mentioned at least one sensation. This may be taken as support for the result that native English speakers scored considerably higher than the Japanese native speakers on Q6 (“I experienced a movement-sensation that I was floating from my real body to the location of the cameras”).

The differences in the introspective reports from the interviews may be related to qualitative differences in the OBEs experienced by English and Japanese native speakers. In other words, when experiencing OBEs, the former is more likely than the latter to experience the perception that their viewpoint and bodily sensations move backward to where the camera is or that their bodies float.

## 4 Discussion

The results of Blocks #1 and #2 indicate that OBEs were induced in both native English and Japanese speakers similarly. These findings are consistent with a previous study by Ehrsson (2007), whose experimental results they replicate. Importantly, no significant differences were found between the English and Japanese native-speaker conditions in NL-Factor. Therefore, the results suggest that people can experience OBEs as a response to similar stimuli, regardless of their native language. Significantly, this is in line with the notion that the OBE is a perceptual-spatial illusion (Ehrsson, 2007; Guterstam and Ehrsson, 2012) and that basic perceptual experiences of the self in space can be reported similarly across native languages. Moreover, our findings have potentially broader implications in the understanding of body ownership.

Several independent studies in Japan, Europe, and the United States have investigated OBEs from the first-person perspective and found similar results on questionnaire ratings (Petkova and Ehrsson, 2008; Kondo et al., 2020). Their results suggest that the reports of full-body ownership have the potential to be similar across native speakers of Japanese and English. Moreover, based on discussions in previous studies (Kilteni et al., 2012; Guy et al., 2023), the sense of embodiment, which refers to the sensations of being inside, having, and controlling a body, arises when the attributes of the virtual body are processed as if they were those of one’s own biological body. This sense of embodiment consists of three subcomponents with sense of self-location, sense of agency, and sense of body ownership. Sense of self-location means the sensation of occupying a specific volume in space; sense of agency means the feeling of possessing overarching motor control; and sense of body ownership means the feeling of ownership of one’s body. Since OBEs are thought to be closely related to these sensations, it is possible that native English speakers and native Japanese speakers perceive these sensations similarly, without significant differences.

As a supplementary discussion of the control questionnaire items in experimental Block #1, a further detailed analysis of the responses might reveal differences between the introspective reports of the two groups. For instance, in experimental Block #1, there was a significant difference in questionnaire item Q6, even though Q6 was only a control questionnaire item. Specifically, native English speakers reported significantly higher scores on the visually related item (Q6). This suggests that they tended to perceive “visual” sensorial changes strongly. These reports were consistent with those recorded only among native English speakers during the unstructured interviews conducted after the completion of all the blocks, wherein all participants were asked: “Please describe, in as much detail as possible, what you experienced and felt during the experiment.” These differences in introspective reports might be related to the qualitative differences in OBEs between native English and Japanese speakers. While it has been reported that cultural differences, including native language, affect human perceptions of the external world (Flecken et al., 2014; Athanasopoulos et al., 2015), our results further suggest that they also affect perceptions of one’s self-body. Although this study used structured questionnaire items previously used by Ehrsson (2007), there may been evaluation limitations in clarifying the effects of cultural differences, including native language, on the perception of the self-body and the relationship between oneself and the world. Further development of the evaluation method, including improvement of the questionnaire items, is required in the future.

The experiment used in this study induced a first-person perspective OBE, but third-person perspective OBEs have also been reported (Lenggenhager et al., 2007). Since bodily sensations change in both cases of first-and third-person OBEs (Moon and Han, 2022), qualitative differences between native English and Japanese speakers may also occur in third-person perspective OBEs. Moreover, previous studies have suggested that full-body illusions are much stronger from the first-person perspective compared to the third-person perspective (Petkova et al., 2011; Maselli and Slater, 2013, 2014; Gorisse et al., 2017). It was a strength that our present study has studied OBE illusion from the first-person perspective. However, further research targeting third-person full-body illusions may need to consider additional items not considered in this study.

This study’s limitations were as follows. First, although this study represents a further step in this field of research, it is necessary to conduct further analyses using larger sample sizes in the future. In Block 2, the experimenter knew the participants’ condition. This could be problematic since the experimenter could inadvertently induce the participant to experience the OBEs or not (e.g., by doing slightly different movements). Therefore, verification through double-blind comparative trials is a necessary future avenue to further this research. Due to constraints in recruiting participants for the experiment, there were differences in the ages of the respective populations of native English and Japanese speakers. The dependence of the results on such age differences will need to be investigated in the future.

In interpreting the results of this study, it is important to note that the participants had some knowledge of the other language, although their proficiency was not perfect. In addition, cultural differences may be more relevant in the interpretation of observed results. Therefore, further experiments are needed in this area, wherein, it will be important to distinguish between the effects of linguistic differences and cultural differences. However, it was apparent in this study that by at least controlling for differences in native language in the experimental conditions, differences could be observed in the verbalization of OBEs.

This study reported that there were no differences in height, weight, or sitting height between the populations of English and Japanese speakers, and did not focus on the influence of physical characteristics of the individuals. On the other hand, previous studies suggested that our perception of body shape influences our body embodiment perception (Warren, 1984; Mark and Vogele, 1987; Warren and Whang, 1987; Proffitt and Linkenauger, 2013). Moreover, modifying morphological aspects of our bodies can consequently impact our perception of the environment’s scale (Stefanucci and Geuss, 2009; Stefanucci and Geuss, 2010; van der Hoort et al., 2011). From the viewpoint of relationships between the perception of body shape and OBEs, further investigation is expected in the future.

As a supplementary argument, we considered the significant difference observed in the control questionnaire items. However, the statements in the control questionnaire items were “weird” and included to control for task compliance, suggestibility and unspecific cognitive bias. Therefore, further detailed analysis of the content of the interview results will be required in the future.

## 5 Conclusion

To investigate whether differences in native language affect people’s ability to undergo scientifically induced OBEs, this study followed the methodology of Experiments #1 and #2 reported by Ehrsson (2007) and invited age-matched native English and Japanese speakers to participate. Thereafter, their OBEs were evaluated using a structured questionnaire based on a visual analog scale. The comparison of the results showed that OBEs were induced in similar ways in both groups, suggesting that people can have OBEs as a response to similar stimuli, regardless of their native language. However, differences in introspective reports between participants suggested that their experiences may differ qualitatively, and this may be due to the different linguistic backgrounds.

## Data availability statement

The raw data supporting the conclusions of this article will be made available by the authors, without undue reservation.

## Ethics statement

The studies involving humans were approved by the Ethics Committee of the Tokyo Institute of Technology. The studies were conducted in accordance with the local legislation and institutional requirements. The participants provided their written informed consent to participate in this study. Written informed consent was obtained from the individual(s) for the publication of any potentially identifiable images or data included in this article.

## Author contributions

HU: Conceptualization, Data curation, Formal analysis, Funding acquisition, Investigation, Methodology, Project administration, Resources, Software, Validation, Visualization, Writing – original draft, Writing – review & editing. YY: Conceptualization, Data curation, Formal analysis, Investigation, Methodology, Software, Validation, Visualization, Writing – review & editing. KU: Conceptualization, Data curation, Formal analysis, Funding acquisition, Investigation, Methodology, Project administration, Resources, Validation, Visualization, Writing – review & editing. YN: Conceptualization, Data curation, Formal analysis, Funding acquisition, Investigation, Methodology, Resources, Validation, Visualization, Writing – review & editing. YM: Conceptualization, Funding acquisition, Methodology, Resources, Supervision, Validation, Writing – review & editing.
