# Supplementary material for: How do differences in native language affect out-of-body experiences?
Source: Front Psychol. 2024 Jun 6;15:1350980. doi: 10.3389/fpsyg.2024.1350980 (PMC11188993; doi:10.3389/fpsyg.2024.1350980)
Supplement: Supplementary file 1 [file Data_Sheet_1.PDF]

Supplementary Material

1 Supplementary Data S1

In the unstructured Interviews, English native speakers were characterized by talking in detail about (1) the sensation of perspective and their body shifting towards the camera and (2) the sensation of body floating or receding. Six of the ten native English speakers mentioned at least one sensation. This may be taken as support for the result that native English speakers scored Q6 ("I experienced a movement-sensation that I was floating from my real body to the location of the cameras") considerably higher than the Japanese ones.

Native English speakers' reports on (1) the sensation of perspective and body shifting towards the camera included the following:

- "I really felt like I was the camera and not like the person."
- "…it felt as if the... the camera's eyes were my eyes."
- "…like I could start losing track of my real body, again, seeing from the camera."
- "I did start to feel like the... like the camera."
- "I was in the camera rather than being sat when the hammer came in the first time, I felt, uh, I was surprised, um, I did feel kind of like the idea that I was in the camera."
- "But throughout the entire thing, I didn't really see me in the chair as me. I sort of figured the camera view was actually where I was physically sitting."

Native English speakers' reference to (2) the sensation of the body floating or receding included the following:

- "So, I was thinking like, oh, I just like floating like a ghost, but I am not."
- "I was kind of floating behind this body, umm, because I felt like I was kind of like an in-between standing-sitting, umm, because the camera felt a little bit like, I did feel a little bit elevated behind myself."

Subsequently, only three out of the nine native Japanese speakers briefly mentioned "the camera's viewpoint" or a "feeling of being on the camera's side," as seen in the three excerpts given below.

Furthermore, none of the Japanese speakers mentioned the feeling of “floating.” The Japanese sentences reported by the native Japanese speakers in the interview are pronounced as follows. For reference, the Japanese sentences themselves are shown in Fig. S2.

- (Pronunciation of Japanese sentences): “dooki no toki ni wa, sono, jibun ga miteiru shiten, kamera no shiten ni jibun no, maa, sonzai ga aru yoo ni kanji te” [“When we were in sync, you know, I felt like I was, well, existing in the perspective that I was looking at, the perspective of the camera.”]

- (Pronunciation of Japanese sentences): “hanmaa no toki wa kekko ushiro ni iru kankaku deshi ta ne. tabun, jikan ga nagai hoo ga ushiro ni iru kankaku no hoo ga tsuyoka tta to omoi masu.” [“I felt like I was behind the hammer. I think I probably had more of a sense of being back there for longer periods of time.”]

- (Pronunciation of Japanese sentences): “me no mae ni iru no ga jibun no karada da na tte omou kanji deha naku te, mushiro, sono, kamera gawa ni iru na tte iu you na kankaku no hoo ga tsuyoka tta ki ga shi masu.” [“I don't feel like it's my body in front of me; it's more like, you know, I feel like I'm on the camera side.”]

The differences in the introspective reports from the interviews may be related to qualitative differences in the OBEs between English and Japanese native speakers. In other words, in undergoing OBEs, the former is more likely than the latter to experience the perception that their viewpoint and bodily sensations move backward to where the camera is or that their bodies float.

2 Supplementary Figures and Tables

2.1 Supplementary Figures

- 「同期の時には、その、自分が見ている視点、カメラの視点に自分の、まあ、存在があるように感じて。」”dooki no toki ni wa, sono, jibun ga miteiru shiten, kamera no shiten ni jibun no, maa, sonzai ga aru yoo ni kanji te” [“When we were in sync, you know, I felt like I was, well, existing in the perspective that I was looking at, the perspective of the camera.”]
- 「ハンマーの時は結構後ろにいる感覚でしたね。多分、時間が長いほうが後ろにいる感覚のほうが強かったと思います。」”hanmaa no toki wa kekko ushiro ni iru kankaku deshi ta ne. tabun, jikan ga nagai hoo ga ushiro ni iru kankaku no hoo ga tsuyoka tta to omoi masu.” [“I felt like I was behind the hammer. I think I probably had more of a sense of being back there for longer periods of time.”]
- 「目の前にいるのが自分の体だなって思う感じではなくて、むしろ、その、カメラ側にいるなっていうような感覚のほうが強かった気がします。」 “me no mae ni iru no ga jibun no karada da na tte omou kanji deha naku te, mushiro, sono, kamera gawa ni iru na tte iu you na kankaku no hoo ga tsuyoka tta ki ga shi masu.” [“I don't feel like it's my body in front of me, it's more like, you know, I feel like I'm on the camera side.”]

Supplementary Figure 2. Sentences with actual Japanese characters, in which actual Japanese speakers briefly mentioned “the camera’s viewpoint” or a “feeling of being on the camera’s side” in the interview for the experiment.
